# A mathematical model for predicting postoperative leg shortening after curved intertrochanteric varus osteotomy for osteonecrosis of the femoral head

**DOI:** 10.1371/journal.pone.0208818

**Published:** 2018-12-18

**Authors:** Tsuyoshi Asano, Daisuke Takahashi, Tomohiro Shimizu, Tohru Irie, Ryuta Arai, Mohamad Alaa Terkawi, Norimasa Iwasaki

**Affiliations:** Department of Orthopedic Surgery, Faculty of Medicine and Graduate School of Medicine, Hokkaido University, Sapporo, Hokkaido, Japan; Harvard Medical School, UNITED STATES

## Abstract

Despite good clinical outcomes associated with curved intertrochanteric varus osteotomy for the treatment of osteonecrosis of the femoral head, post-operative leg-length discrepancy is frequently reported and might reduce patient satisfaction. Although previous report showed that varus angulation affected post-operative leg-length discrepancy, sufficient varus angulation is the most important factor for obtaining a lateral intact portion. Therefore, to ensure better postoperative outcomes, detection of other parameters associated with leg shortening may prove useful. This study aimed to detect other factors influencing post-operative leg-length discrepancy and to develop a theory for pre-operative planning. The study included 42 hips of 36 patients with osteonecrosis of the femoral head [25 men and 11 women; mean age at the time of surgery, 33.8 years (range, 17 to 53 years)]. Patients were assessed their clinical and radiological results bofore and after surgery. Additionally, a mathematical model was developed to predict leg shortening after curved intertrochanteric varus osteotomy based on the degree of varus angulation and the distance between the femoral head and osteotomy arc centers. Predicted and actual leg shortening in patients were compared to verify the accuracy of our model. Post-operatively, mean varus angle was 21.7° (range, 15 to 38°) and mean leg shortening was 1.7 mm (range, -5.1 to 11.4 mm). Univariate analysis showed that varus angulation and lateral shift of the osteotomy arc might influence the degree of leg shortening. Furthermore, mathematically predicted leg shortening significantly correlated with actual leg shortening (r = 0.905, p < 0.001), suggesting the usefulness of our model for predicting complications of curved intertrochanteric varus osteotomy. This study indicates the importance of not positioning the center of the osteotomy arc lateral from the center of the femoral head to minimize leg shortening after curved intertrochanteric osteotomy.

## Introduction

Osteonecrosis of the femoral head (ONFH) is one of the leading causes of femoral head collapse and hip dysfunction [1, 2]. It has been reported that ONFH is the most common cause of total hip replacement (THR) in young individuals [3]. However, THR is not the best option for young patients and is often associated with complications that lead to revision surgery [4, 5]. Femoral osteotomy, including varus osteotomy, involves preserving procedures aimed at avoiding or delaying THR. In these procedures, surgeons move the necrotic region of the femoral head and place the intact region into a weight-bearing position.

Curved intertrochanteric varus osteotomy (CVO) has been described for the treatment of osteoarthritis of the dysplastic hip and is applied for ONFH [6]. This osteotomy procedure is performed between the greater and lesser trochanters to place the femoral head in a varus position. Several studies reported that CVO leads to good clinical results, preventing collapse and delaying THR for the treatment of ONFH [7–11]. However, post-operative leg shortening often occurs at an average of 10–13 mm in the majority of patients. Although previous reports showed that varus angulation affected post-operative leg-length discrepancy [8], sufficient varus angulation is the most important factor for obtaining a lateral intact portion [12, 13]. Therefore, to ensure better postoperative outcomes, it might be useful and important to detect other parameters associated with post-operative leg shortening.

The objectives of this study were to detect other factors influencing post-operative leg-length discrepancy and to develop a theory for pre-operative planning for CVO. Because several surgical devices have recently been developed to ensure better accuracy of CVO, pre-operative planning to reduce post-operative leg shortening could lead to improved clinical results in patients receiving CVO.

## Materials and methods

### Patients

**Fig 1:**
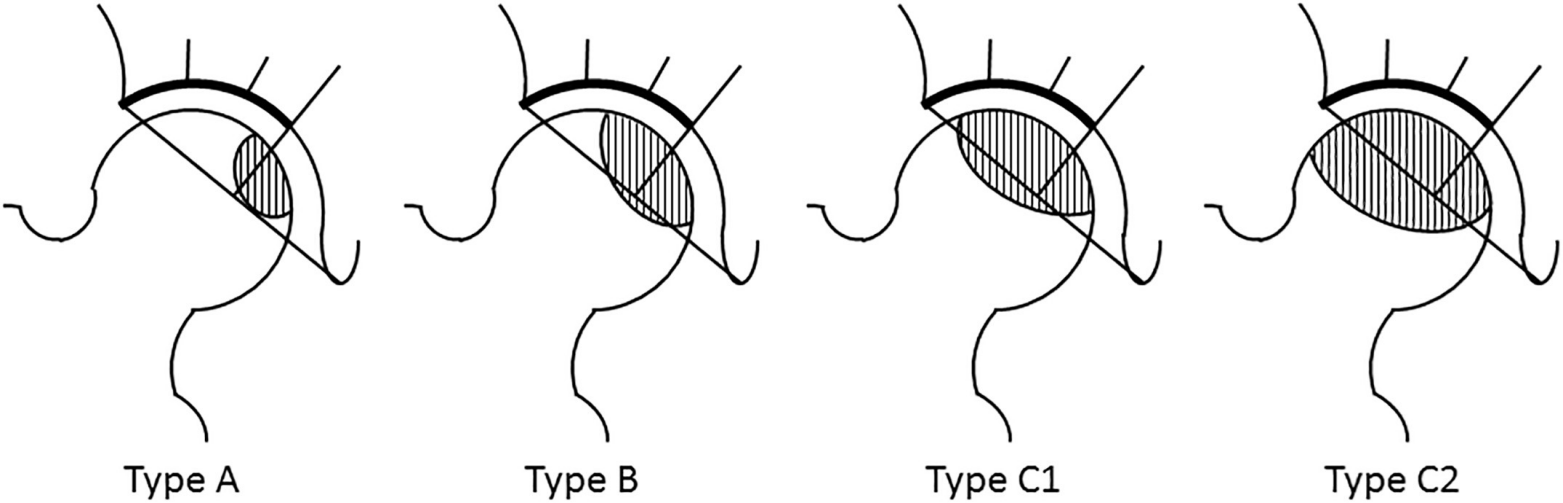
The classification of osteonecrosis of femoral head (Japanese Investigation Committee of Health and Welfare).

Between November 2010 and March 2016, 42 CVOs were performed to treat ONFH in 36 patients. Six patients had bilateral operations. The patients included 25 males and 11 females, with a mean age at the time of surgery of 33.8 years (range, 17 to 53 years). The mean follow-up period was 32 months (range, 12 to 76 months). The diagnosis of osteonecrosis was made based on clinical history, physical examination, and imaging studies including radiographs and magnetic resonance imaging [14]. Cases of osteonecrosis were categorized as corticosteroid-associated in 22 hips, alcohol-associated in 18 hips, and idiopathic in 2 hips. They were staged according to Association Research Circulation Osseous (ARCO) staging system and classified according to the classification of the Japanese Investigation Committee of Health and Welfare [15]. Fig 1 showed the classification of the ONFH in this study. Necrotic lesions are classified into four types, based on their location on T1- weighted images or x-ray images. Type A lesions occupy the medial one-third or less of the weight-bearing portion. Type B lesions occupy the medial two-thirds or less of the weight-bearing portion. Type C1 lesions occupy more than the medial two-thirds of the weight-bearing portion but do not extend laterally to the acetabular edge. Type C2 lesions occupy more than the medial two-thirds of the weight-bearing portion and extend laterally to the acetabular edge (Fig 1). Table 1 summarized the staging and classification in this study. More specifically, 10 hips were staged as stage 2, 28 as stage 3A, and 4 as stage 3B. And the localization of the necrotic lesion was noted to be type C1 in 33 hips and type C2 in 9 (Table 1). The protocol of this retrospective study was approved by institutional review board of Hokkaido University Hospital (approval number: 015–0207) and informed consent were obtained from all patients or their parents when they were younger than 20 years old.

**Table 1:** Patient characteristics.

Characteristics	Value
Age (year) (range)	33.8 (17 to 53)
Gender (hips, %)	
Male	25 patients, 29 hips (69)
Female	11 patients, 13 hips (31)
Body mass index (kg/m^2^) (range)	23.3 (16.1 to 31.2)
Etiology (hips, %)	
Steroid	22 (52)
Alcohol	17 (40)
Idiopathic	3 (7)
Stage (hips, %)	
2	10 (24)
3A	28 (67)
3B	4 (10)
Type (hips, %)	
B	0 (0)
C-1	33 (79)
C-2	9 (21)

### Indication and surgical procedure

**Fig 2:**
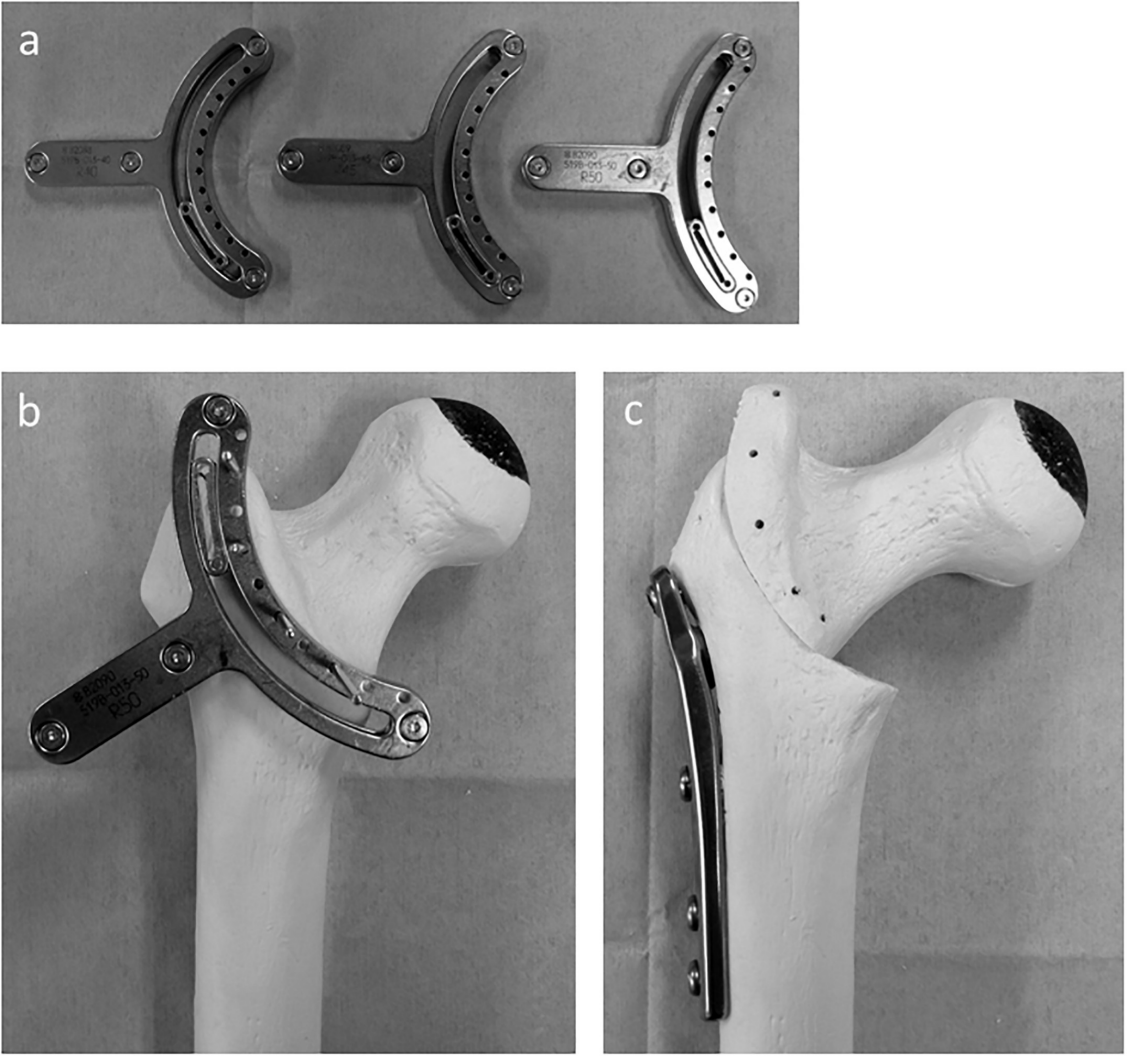
Photographs of curved intertrochanteric varus osteotomy model. a) The crescentic osteotomy guides. The diameters of the osteotomy arc were 40 mm (left), 45 mm (center), and 50 mm (right), respectively. B) The osteotomy guide is fixed with three to four guide pins; osteotomy was carried out using a reciprocating saw along with the sliding slit of the guide. C) Internal fixation after cutting and varus angulation was performed with a compression hip screw system. The intact lateral region becomes the new weight-bearing portion.

Indication for CVO was decided from age (55 years younger), the joint-sparing hope and the viable portion of femoral head. It has been reported that the clinical result of femoral osteotomy depends on the area of the intact articular surface on the femoral head post-operatively [9, 12]. The lateral head index (LHI) has been used as a radiographic parameter for assessing the area of the intact portion [16]. LHI with an intact area greater than 25% was considered a good clinical outcome [13], and osteotomy was indicated for patient with LHI ≥ 25% at maximum abduction pre-operatively. The surgical procedure and post-operative regimen were carried out as described previously [7]. Briefly, after the lesser trochanter and inter-trochanteric crest were exposed, the crescentic guide (Meira, Aichi, Japan) was attached at least 5 mm lateral to the inter-trochanteric crest to preserve the posterior retinacular artery (Fig 2a and 2b). Then, the osteotomy was carried out using a reciprocating saw. After detaching the iliopsoas tendon, the proximal fragment was moved into varus, and the planned varus angle was confirmed by an image intensifier, measuring the distance that the fragments had been moved. The osteotomy was stabilized with a compression hip screw system designed for varus osteotomy (Meira) (Fig 2c). The post-operative regimen allowed the patients to perform partial weight-bearing after 4 weeks and full weight-bearing after 8 weeks.

### Assessments

**Fig 3:**
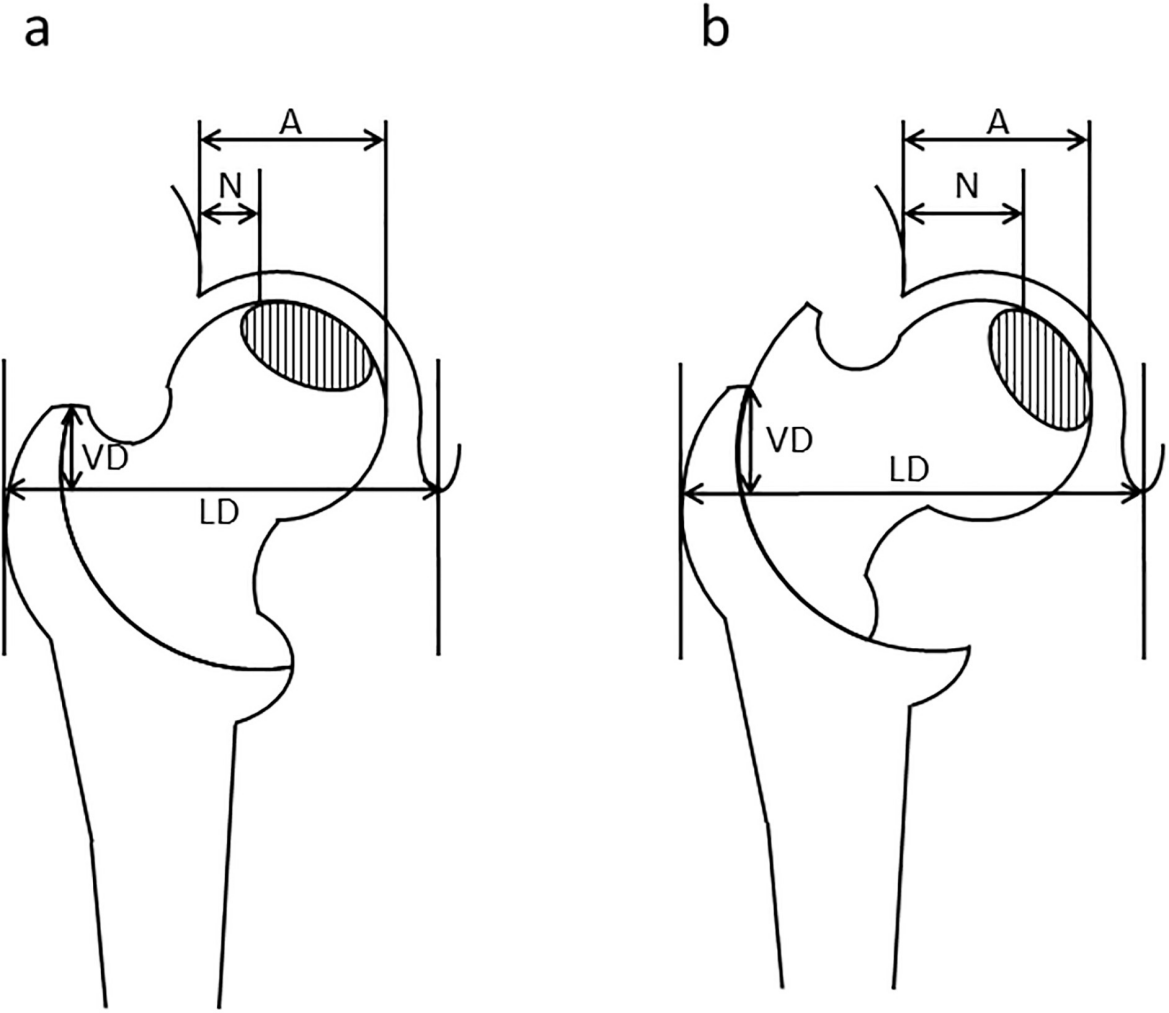
Diagram for the measurement of leg shortening and lateralization and the lateral head index (LHI). a) Before and b) after osteotomy. Shaded area represents the necrotic region. LHI was calculated as the width of the intact region (N) divided by the full length of the femoral head (A) and expressed as a percentage.

**Fig 4:**
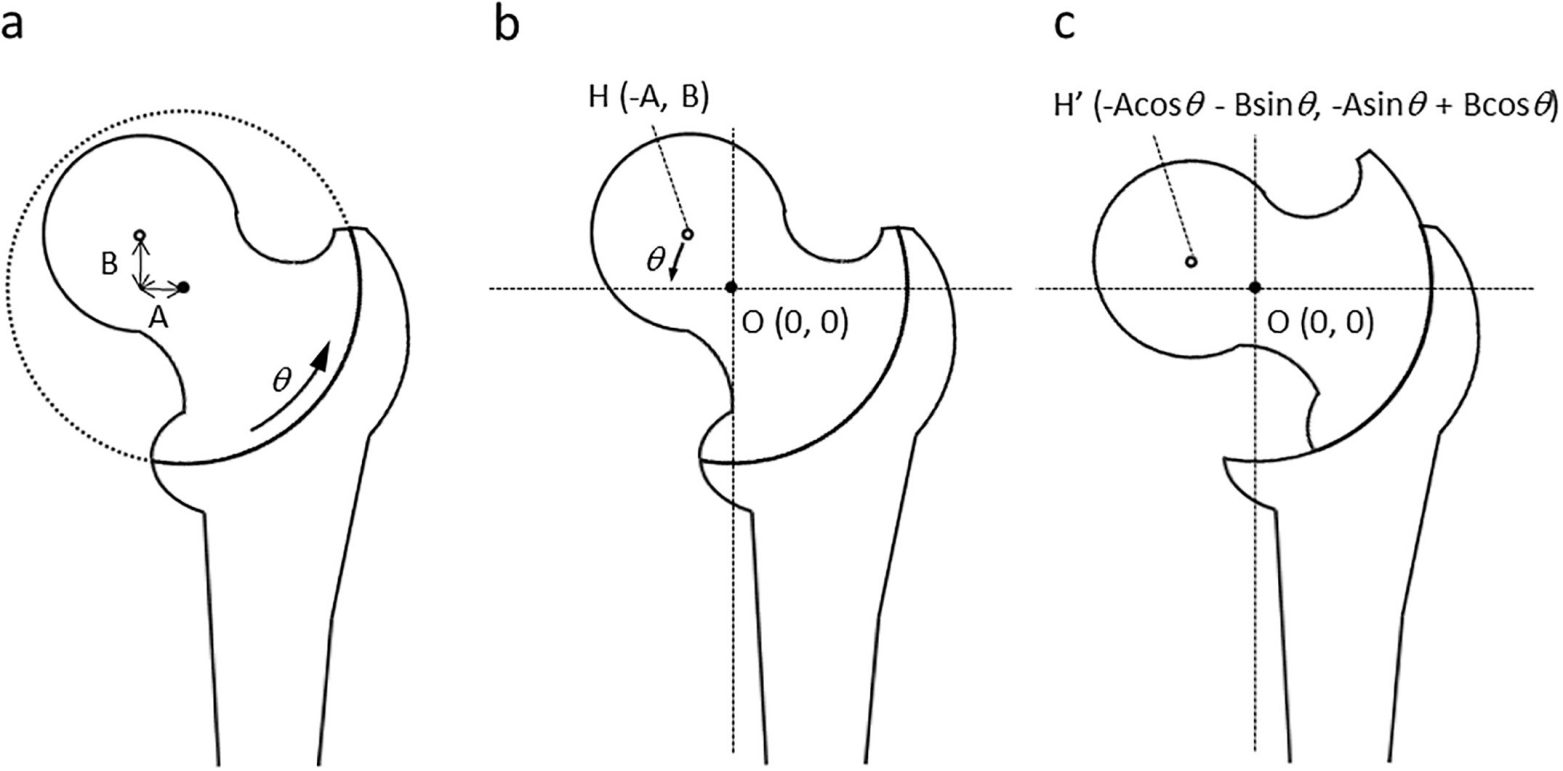
Diagram of mathematical model of curved intertrochanteric varus osteotomy. a) Black points show the center of the osteotomy arc (O), defined as a origin (0, 0). White points indicate the center of the femoral head (H). The lateral shift and distal shift of the center of the femoral head from origin are A and B, respectively. B) Before and c) after osteotomy; the coordinate of H changes as shown.

Clinical evaluation was performed using the Harris Hip Score (HHS) pre-operatively and during follow-up [17]. Anteroposterior (AP) radiographs of the hip in the neutral position obtained before and after surgery were analyzed to calculate varus angulation, pre- and post-operative LHI, shortening and lateralization of the femur, center of the femoral head, and the osteotomy arc. Varus angulation was calculated by subtracting the post-operative neck-shaft angle from the pre-operative angle. To clarify the area of necrotic portion, gadolinium-contrast MRI were performed pre-operatively. LHI was measured as described (Fig 3). Additionally, to measure LHI in maximal abduction preoperative, we firstly measured the area of necrotic portion at neutral position by gadolinium-contrast MR-imaging and plotted this area to the plain radiograph with neutral position. After obtained plain radiograph with maximal abduction and same rotation as neutral position, LHI in maximal abduction was measured by superimposition to plotted image. The vertical distance (VD) from the proximal end of the greater trochanter to the tear-drop line and the lateral distance (LD) from the tear-drop line to the lateral border of the greater trochanter were measured. Leg shortening was expressed by subtracting pre-operative from post-operative VD. The lateralization of the femur was expressed by subtracting pre-operative from post-operative LD. The centers of the femoral head and osteotomy arc were determined from post-operative radiographs. Imaging software was used to determine the distance of the center of the femoral head from three designated points of the femoral head and from the arc. The lateral shift (A) and distal shift (B) of the center of the femoral head from the center of the osteotomy arc were measured (Fig 4a).

### Mathematical model

The center of the osteotomy arc was defined as (O) origin (0, 0), and that of the femoral head (H) was defined as (-A, B) pre-operatively (Fig 4b). Adding the rotation of varus angulation of *θ*, the post-operative coordinates of the center of the femoral head (H’) were (-Acos*θ*-Bsin*θ*, -Asin*θ*+Bcos*θ*) (Fig 4c). It is worth noting that the relation between the distal femoral fragment and the center of the osteotomy arc does not change before and after osteotomy. The theoretical leg shortening was determined by subtracting the pre-operative from the post-operative vertical coordinate of the femoral head (Leg shortening = Asin*θ* + B(1-cos*θ*)). The speculative lateralization was also determined by subtracting the pre-operative from the post-operative horizontal coordinates (Femoral lateralization = -A(1-cos*θ*) + Bsin*θ*).

The correlation between actual and speculative leg shortening and lateralization were analyzed using Pearson’s correlation coefficient. To evaluate the intra-rater and inter-rater agreement, intra-class and inter-class correlation coefficient were calculated. Statistical analyses were performed using SPSS software v.19.0 (SPSS Inc. Chicago, IL, USA).

## Results

All treated patients had no conversion to THR or post-operative complications such as infection, fracture, or nonunion over the follow-up period. The mean pre-operative HHS was 63.2 (range, 32 to 86), and it improved significantly to 93.6 (58 to 100) at the end of the follow-up period. The mean varus angulation obtained by the osteotomy was 21.7° (15 to 38°). Consistently, LHI increased from 11.8% (0% to 26%) to 36.7% (21% to 53%) after osteotomy. Actual leg shortening was determined to be 1.7 mm (-5.1 to 11.4 mm), and actual femoral lateralization was -1.1 mm (-11.5 to 8.2 mm).

**Fig 5:**
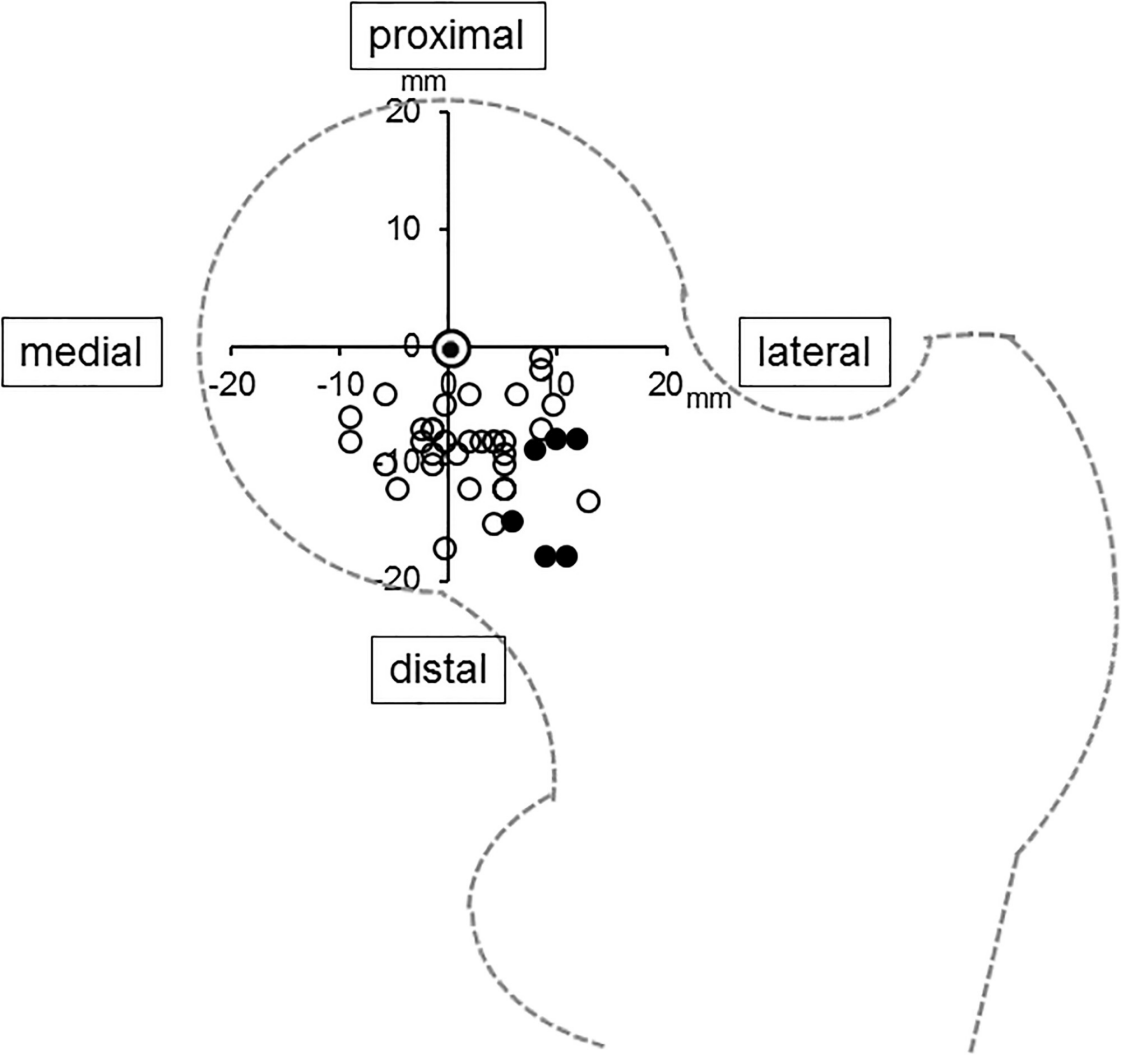
Scatter diagram for the distribution of the center of the osteotomy arc compared to the center of the femoral head. Double circle indicates the center of the femoral head. White circles indicate the centers of the osteotomy arc of cases with leg shortening < 5mm. Black circles indicate the centers of the osteotomy arc of cases with leg shortening ≥ 5mm.

Only 7 cases (17%) showed ≥ 5 mm leg shortening. The lateral shift (A) and distal shift (B) of the center of the osteotomy arc from the center of the femoral head were 3.3 mm (-8 to 21 mm) and 9.3 mm (1 to 21 mm), respectively (Table 2). In all hips, the center of the osteotomy arc was positioned to the distal side of the center of the femoral head. The distribution of the center of the osteotomy arc compared to the center of the femoral head is shown in a scatter diagram (Fig 5). There was significant correlation between varus angulation and leg shortening (r = 0.391, p < 0.01). Additionally, there was significant correlation between the lateral shift of the center of the osteotomy arc from the femoral head and leg shortening (r = 0.839, p < 0.01). Intra-class and inter-class correlation coefficients (ICC) were almost excellent (intra: 0.907, p = 0.006 and inter: 0.793, p = 0.017, respectively).

**Table 2:** Clinical and radiographic results.

Characteristics	Value
Harris Hip Score	
Pre-operative	63.2 (32 to 86)
Post-operative	93.6 (58 to 100)
Duration of follow-up (month)	36.0 (15 to 80)
Lateral Head Index (%)	
Pre-operative	11.8 (0 to 26)
Post-operative	36.7 (21 to 53)
Varus angulation (degree)	21.7 (15 to 38)
Actual leg shortening (mm)	1.7 (-5.1 to 11.4)
Actual lateralization (mm)	-1.1 (-11.5 to 8.2)
Shift of the center of the osteotomy arc from femoral head	
Lateral shift (A) (mm)	3.3 (-8.0 to 21.0)
Distal shift (B) (mm)	9.3 (1.0 to 21.0)

**Fig 6:**
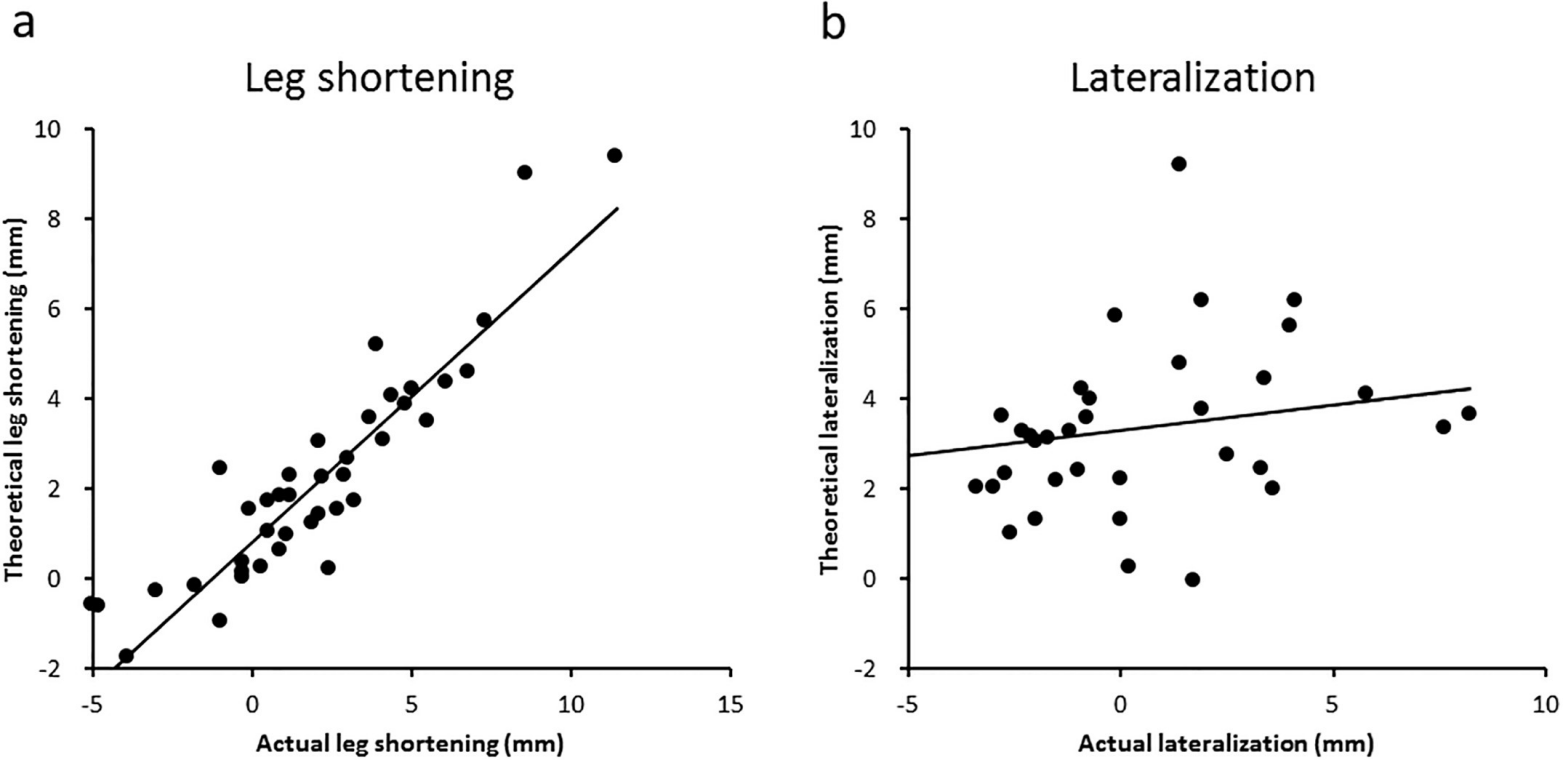
Correlation of the actual and theoretical changes after curved intertrochanteric varus osteotomy. a) Correlation of the actual and theoretical leg shortening and b) correlation of the actual and theoretical lateralization are shown.

**Fig 7:**
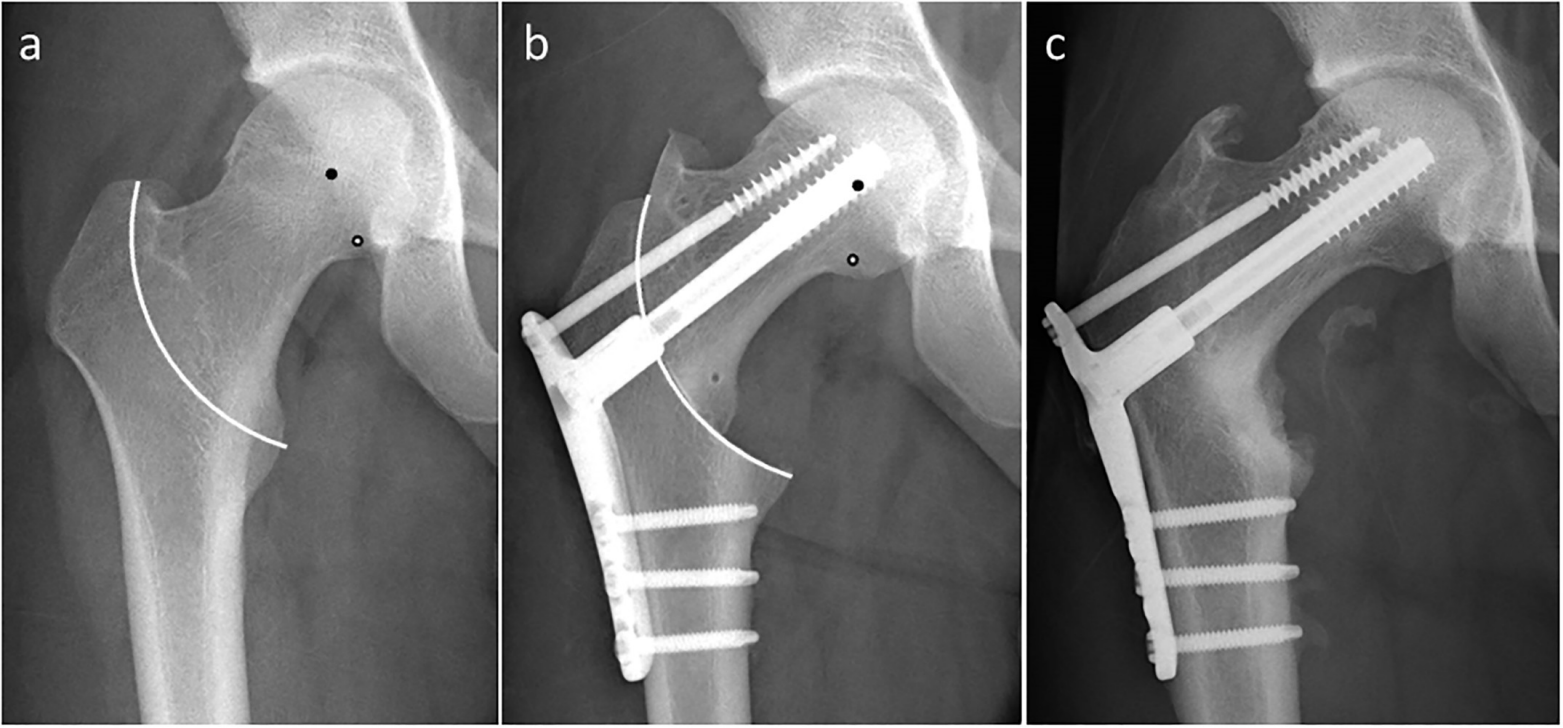
Radiographs of 20-year-old man who had steroid-associated osteonecrosis of the femoral head treated with curved intertrochanteric varus osteotomy. a) Before and b) after the operation. White line indicates the osteotomy arc. Black points indicate the center of the femoral head and white points indicate the center of the osteotomy arc. The actual leg shortening was -2.0 mm, whereas the theoretical leg shortening was -1.6 mm.

**Fig 8:**
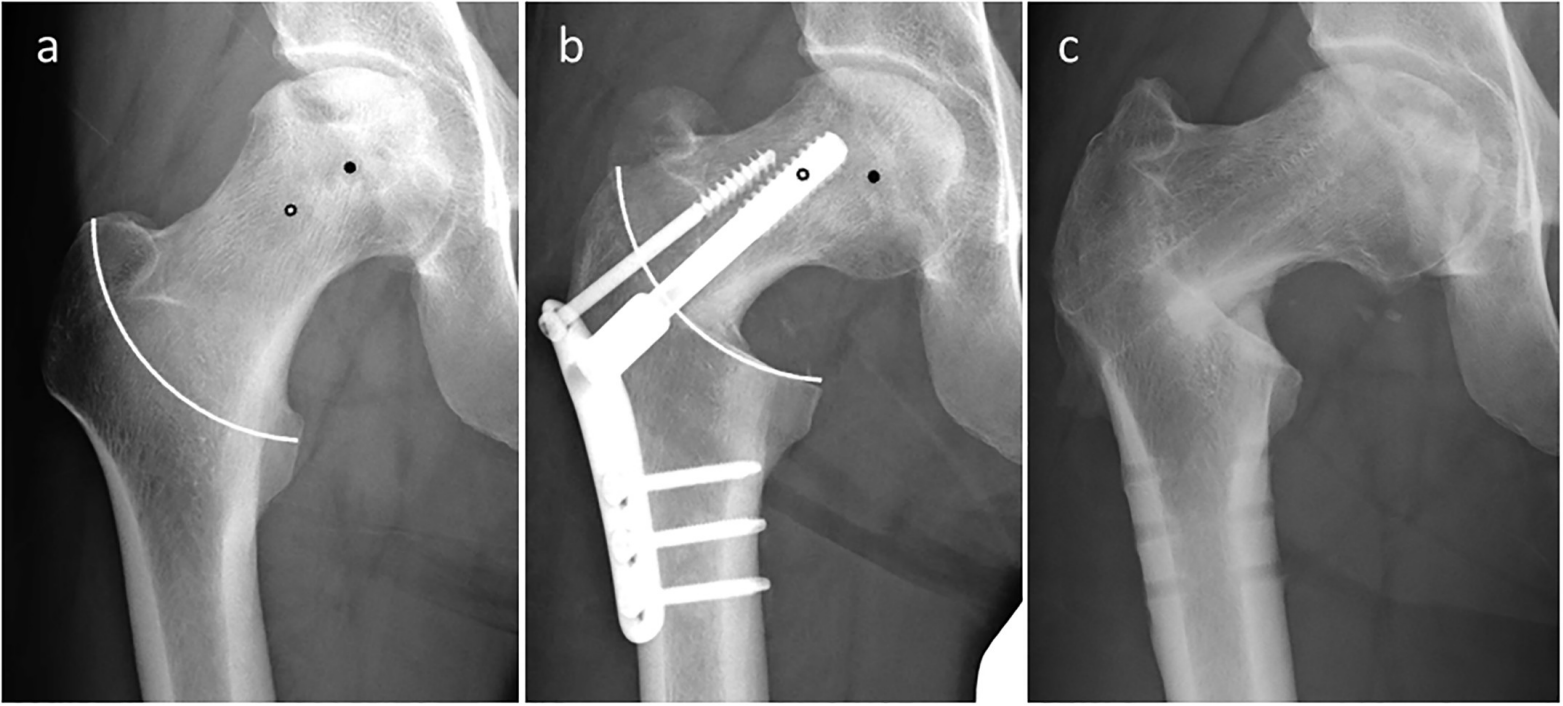
Radiographs of 39-year-old man who had steroid-associated osteonecrosis of the femoral head treated with curved intertrochanteric varus osteotomy. a) Before and b) after the operation. White line indicates the osteotomy arc. Black points indicate the center of the femoral head and white points indicate the center of the osteotomy arc. The actual leg shortening was 8.6 mm, whereas the theoretical leg shortening was 9.0 mm.

Based on the mathematical model, we calculated the theoretical leg shortening and femoral lateralization using varus angulation, lateral shift, and distal shift. The predicated leg shortening and lateralization obtained by the mathematical model were 2.0 mm (-3.0 to 9.4 mm) and 3.2 mm (0.0 to 9.2 mm), respectively. There was a strong correlation between actual leg shortening and theoretical leg shortening (r = 0.905, p < 0.001). On the other hand, actual femoral lateralization showed a weak correlation with theoretical femoral lateralization (Fig 6). Representative radiographs are shown in Fig 7. If the center of the osteotomy arc was located distal but not lateral to the center of the femoral head, leg shortening did not occur. On the other hand, if the center of the osteotomy arc was located dislat and lateral to the center of the femoral head, leg shortening occurred (Fig 8). At the final follow up, anterior impingement sign and osseous bump formations on the edge of the femoral head were observed in 6 hips of 42 hips.

## Discussion

In this study, we investigated the associations between radiographic changes and post-operative shortening, and developed a theory for pre-operative planning. CVO was developed for the prevention of leg shortening, elevation of the greater trochanter, and lateral displacement of the femoral shaft associated with other osteotomy procedures. It was reported that CVO resulted in good mid- and long-term outcomes in terms of avoiding THR. Zhao et al. [9] reported that 91.8% of hips remained intact without conversion to THR at a mean follow up of 12.4 years. Okura et al. [11] reported a 10-year survival rate of 91.0% with conversion to THR as the endpoint. However, approximately 10 mm of leg shortening after CVO still remained in the studies. Therefore, there is a need to improve this procedure in order to reduce leg shortening.

There is little data concerning factors affecting leg shortening after CVO. Consistent with a previous report [8], the current study showed that varus angulation correlated with post-operative leg shortening. Increasing the varus angulation to obtain a greater lateral intact portion may result in a higher risk of leg shortening. However, successful femoral osteotomy for treatment of ONFH is essentially dependent on the intact portion of the femoral head after osteotomy [18–20]. Leg shortening often occurs due to elevation of varus angulation to obtain a greater lateral intact portion [13]. Therefore, it is important to define factors other than varus angulation affecting leg shortening. The findings of this study also show that there was significant correlation between the lateral shift of the center of the osteotomy arc from the center of the femoral head and post-operative leg shortening, suggesting that it is important to avoid locating the center of the osteotomy arc far lateral from the center of the femoral head during pre-operative planning.

Theoretically, if the centers of the femoral head and osteotomy arc are completely matched, there is no leg shortening or lateral displacement of the femoral shaft at any varus position. However, it seems difficult to locate the center of the osteotomy arc on the center of the femoral head because of the anatomy of the proximal femur. Although Ikemura et al. [8] described that this difference between the centers of the femoral head and osteotomy arc might be the etiology of leg shortening, there was no consensus on the osteotomy location for CVO that leads to minimum leg shortening. In the mathematical calculation model of this study, predicated leg shortenings strongly correlated with actual leg shortening, indicating the usefulness of this model. In this study, we performed CVO based on our mathematical model, and the average observed post-operative leg shortening was 1.7 mm, representing good post-operative results. In contrast, only a weak correlation between theoretical and actual femoral lateralization was obtained. This can be explained by adduction/abduction and rotation of the hip joint that may influence the lateral distance from the tear drop line to the greater trochanter. However, because leg shortening is clinically more important than femoral lateralization [13], the present mathematical model is thought to be sufficiently useful.

This study has some limitations. First, CVO is not a common surgical procedure worldwide. However, we believe that CVO could have wide surgical indications for ONFH to prevent collapse and delay THR, as well as core decompression and free vascularized fibular grafting. In addition to recent developments in surgical devices, this theory might potentially provide a safe and reliable surgical framework. Second, this study had a small sample size and short observation period for the evaluation of clinical outcomes and the survivals of hips in patients post-operatively. However, since this model led to a lateral intact portion, which was similar to earlier reports, we expect promising long-term outcomes.

## Conclusions

This study indicates the importance of not positioning the center of the osteotomy arc lateral to the center of the femoral head, in order to minimize leg shortening after curved intertrochanteric osteotomy. Leg shortening after CVO can be reduced with pre-operative planning based on the presented mathematical model.
